# Supplementary material for: The Use of Social Media for Preconception Information and Pregnancy Planning among Young Women
Source: J Clin Med. 2021 Apr 27;10(9):1892. doi: 10.3390/jcm10091892 (PMC8123806; doi:10.3390/jcm10091892)
Supplement: Supplementary file 1 [file jcm-10-01892-s001.zip › jcm-1176908-supplementary.pdf]

File S1-SURVEY QUESTIONS

Social media use for preconception and pregnancy information among young women of reproductive age

1. ***What is your age in years?***

2. ***What is your level of education studied or currently studying?***
- Higher University Degree (e.g. Grad Dip, Masters, PhD)
- Bachelor Degree
- Advanced Diploma
- Certificate Level
- Year 12 or equivalent
- Did not finish Secondary School

3. ***What is your relationship status?***
- Married
- Partnered
- Single

4. ***What is your employment status?***
- Full Time
- Part Time
- Casual
- Not working

5. ***What is your annual individual income?***
- $1-$6,239 annually
- $6,240-$15,999 annually
- $16,000-$25,999 annually
- $26,000-$36,999 annually
- $37,000-$51,999 annually
- $52,000 + annually
- I'd rather not answer
- No Income

6. ***Do you have or are you currently caring for children (e.g. as a parent or step parent)?***
- No
- Yes

7. ***Are you planning a pregnancy in the near future?***

- No,
    - If no, please specify why
- Yes, within the next 6-12 months
- Yes, within the next 12 months- 5 years

In this section, we explore information related to Social Media use.

8. ***Do you feel you have adequate knowledge about pregnancy from your past or current education?***

- Extremely adequate knowledge
- Somewhat adequate knowledge
- Neither adequate nor inadequate knowledge
- Somewhat inadequate knowledge
- Extremely inadequate knowledge

Please explain your reasoning for the above answer

9. ***Do you use a particular app or social media platform to gain information about your overall health?***

- No
- Yes

10. **Please indicate how often you use the following social media platforms/apps to gain information about you overall health**

Instagram - overall health

- Daily
- About once a week
- 2-3 times a week
- 4-6 times a week
- About once a month
- Never - I do not use Instagram for information about my overall health.

Facebook - overall health

- Daily
- About once a week
- 2-3 times a week
- 4-6 times a week
- About once a month
- Never - I do not use Facebook for information about my overall health.

SnapChat Stories - overall health

- Daily
- About once a week
- 2-3 times a week
- 4-6 times a week
- About once a month
- Never - I do not use SnapChat for information about my overall health.

Twitter- overall health

- Daily
- About once a week
- 2-3 times a week
- 4-6 times a week
- About once a month
- Never - I do not use Twitter for information about my overall health.

Tumblr - overall health

- Daily
- About once a week
- 2-3 times a week
- 4-6 times a week
- About once a month
- Never - I do not use Tumblr for information about my overall health.

Other (please specify) - overall health

- Daily
- About once a week
- 2-3 times a week
- 4-6 times a week
- About once a month

11. ***Do you use social media platforms/ apps to seek preconception-health-related information?***

- Yes
- No

12. **Please indicate how often you use the following social media platforms/ apps to engage in preconception-health-related information**

Instagram - preconception-health-related information

- Daily
- About once a week
- 2-3 times a week
- 4-6 times a week
- About once a month
- Never - I do not use Instagram for information about preconception health.

Facebook - preconception-health-related information

- Daily
- About once a week
- 2-3 times a week
- 4-6 times a week
- About once a month
- Never - I do not use Facebook for information about preconception health.

SnapChat Stories - preconception-health-related information

- Daily
- About once a week
- 2-3 times a week
- 4-6 times a week
- About once a month
- Never - I do not use SnapChat for information about preconception health.

Twitter - preconception-health-related information

- Daily
- About once a week
- 2-3 times a week
- 4-6 times a week
- About once a month
- Never - I do not use Twitter for information about preconception health.

Tumblr** - **preconception-health-related information

- Daily
- About once a week
- 2-3 times a week
- 4-6 times a week
- About once a month
- Never - I do not use Tumblr for information about preconception health.

Other (please specify) - preconception-health-related information

- Daily
- About once a week
- 2-3 times a week
- 4-6 times a week
- About once a month

13. Would you use social media platforms/apps to engage in preconception health-related information? (e.g in the future)

- Yes
- No

14. Which platforms you would use to seek preconception health-related information in the future?

15. Do you share preconception health-related information from social media pages with your peers?

- Yes
- No

16. Please describe in what situation you share preconception health-related information with your peers (e.g. sharing on Facebook, retweeting etc.).

17. Do you use social media platforms/ apps to engage in and seek pregnancy related information?

- Yes
- No

18. Please indicate how often you use the following social media platforms/ apps to seek pregnancy- related information

Instagram - pregnancy-health-related information

- Daily
- About once a week
- 2-3 times a week
- 4-6 times a week
- About once a month
- Never - I do not use Instagram for information about pregnancy health.

Facebook - pregnancy-health-related information

- Daily
- About once a week

- 2-3 times a week
- 4-6 times a week
- About once a month
- Never - I do not use Facebook for information about pregnancy health.

SnapChat Stories - pregnancy-health-related information

- Daily
- About once a week
- 2-3 times a week
- 4-6 times a week
- About once a month
- Never - I do not use SnapChat for information about pregnancy health.

Twitter - pregnancy-health-related information

- Daily
- About once a week
- 2-3 times a week
- 4-6 times a week
- About once a month
- Never - I do not use Twitter for information about pregnancy health.

Tumblr – pregnancy-health-related information

- Daily
- About once a week
- 2-3 times a week
- 4-6 times a week
- About once a month
- Never - I do not use Tumblr for information about pregnancy health.

Other (please specify) - pregnancy-health-related information

- Daily
- About once a week
- 2-3 times a week
- 4-6 times a week
- About once a month

19. ***Would you use social media platforms/apps to engage in pregnancy health-related information? (e.g in the future)***

- Yes
- No

20. ***Which platforms you would use to seek pregnancy health-related information in the future?***

21. ***Do you share pregnancy health-related information from social media pages with your peers?***

- Yes
- No

22. ***Please describe in what situation you share pregnancy health-related information with your peers (e.g. sharing on Facebook, retweeting etc.).***
